# Fully endoscopic transforaminal discectomy for thoracolumbar junction disc herniation with or without calcification under general anesthesia: Technical notes and preliminary outcomes

**DOI:** 10.3389/fsurg.2022.1067775

**Published:** 2023-01-06

**Authors:** Shengwei Meng, Jialuo Han, Derong Xu, Yan Wang, Shuo Han, Kai Zhu, Antao Lin, Kunpeng Su, Yaxiong Li, Xing Han, Xuexiao Ma, Chuanli Zhou

**Affiliations:** ^1^ Department of Spinal Surgery, The Affiliated Hospital of Qingdao University, Qingdao, China; ^2^ Operating Room, The Affiliated Hospital of Qingdao University, Qingdao, China

**Keywords:** endoscopic spinal surgery, transforaminal, discectomy, ventral decompression, thoracolumbar junction zone, intervertebral disc displacement

## Abstract

**Objective:**

To evaluate the feasibility, safety, and outcomes of percutaneous endoscopic transforaminal discectomy (PETD) for thoracolumbar junction disc herniation (TLDH) with or without calcification.

**Methods:**

This study included 12 patients diagnosed with TLDH with or without calcification who met the inclusion criteria and underwent surgery for PETD from January 2019 to December 2021. The mean patient age, operation time, hospitalization time, time in bed, and complications were recorded. Patients were followed up for at least 9 months. Visual analog scale (VAS) scores for low-back and leg or thoracic radicular pain and modified Japanese Orthopedic Association score (m-JOA) scores were preoperatively evaluated, at 1 day and 3, 6, and 12 months postoperatively or at last follow-up. The modified MacNab criteria were used to evaluate clinical efficacy at 12 months postoperatively or at last follow-up.

**Results:**

The mean patient age, operation time, hospitalization time, and time in bed were 53 ± 13.9 years, 101.3 ± 9.2 min, 4.5 ± 1.3 days, and 18.0 ± 7.0 h, respectively. The mean VAS scores of low-back and leg or thoracic radicular pain improved from 5.8 ± 1.5 and 6.5 ± 1.4 to 2.0 ± 0.9 and 1.3 ± 0.5, respectively (*P *< 0.05). The m-JOA score improved from 7.5 ± 1.2 to 10.0 ± 0.7 (*P* < 0.05). The overall excellent–good rate of the modified MacNab criteria was 83.3%. No severe complications occurred.

**Conclusion:**

Fully endoscopic transforaminal discectomy and ventral decompression under general anesthesia is a safe, feasible, effective, and minimally invasive method for treating herniated discs with or without calcification at thoracolumbar junction zone.

## Introduction

The thoracolumbar junction usually refers to the region from T11 to L2 in clinical practice (1). Thoracolumbar junction disc herniation (TLDH) with an incidence of <5% of all lumbar disc herniations is much less common than in the lower cervical and lower lumbar spines (1–4). However, TLDH is sometimes encountered in our clinical practice. Generally, the risks of surgical operation at the thoracolumbar junction zone are greatly increased because the spinal canal at these levels accommodates the spinal cord, conus medullaris, or cauda equina. Additionally, clinical manifestations of TLDH are complex and various, including low back pain, intercostal neuralgia, leg pain, groin region pain, lower limb numbness with or without weakness, and walking difficulty, which causes severe suffering for patients (1). Moreover, postoperative TLDH outcome is worse than lower lumbar disc herniation (5). The classical posterior approach, including laminectomy and discectomy with or without internal fixation, requires extensive paravertebral muscle and facet joint resection to fully expose the herniated disc and dura sac, leading to spinal instability and leaving the patient susceptible to persistent low back pain and a higher risk of nerve injury (6, 7).

Nowadays, percutaneous endoscopic discectomy is well accepted by surgeons and patients for cervical and lumbar disc herniation treatment because of advantages like less trauma, less bleeding, faster recovery, and lower complication rates (8). Percutaneous endoscopic discectomy and decompression were introduced for treating thoracic disc herniation and thoracic stenosis with advances in endoscopic visualization and instrumentation (9, 10). However, percutaneous endoscopic transforaminal discectomy (PETD) for TLDH is rarely reported. Thus, this study performed a fully endoscopic transforaminal ventral discectomy technique, PETD, to treat patients with thoracolumbar junction zone disc herniation. This paper reports our technical notes of fully endoscopic transforaminal ventral discectomy for TLDH and the preliminary outcomes of 12 cases.

## Materials and methods

### Participants

We treated 15 patients diagnosed with TLDH using PETD from January 2019 to December 2021; of them, 12 met the inclusion criteria. More than one spinal surgeon was invited to diagnose based on clinical manifestations and imaging findings. All surgeries were completed by two skilled surgeons with extensive experience in the endoscopic technique. Table 1 shows the patients’ clinical characteristics. All procedures were authorized by the ethics committee of our institution. Written informed consent was obtained from all included patients. The privacy and critical interests of our patients were protected following the Declaration of Helsinki.

**Table 1:** Demographic findings of the study patients (*n* = 12).

Characteristic	Mean ± SD or *n*
Age (years)	53 ± 13.9
Sex, male:female	7:5
Side of the surgery, left:right	5:7
Levels involved, T11–12:T12–L1:L1–2	4:5:3
Low back pain	9
Leg pain	7
Thoracic radiculopathy	5
Paresthesia in lower limb	8
Lower limb weakness	5
Neurogenic claudication	7
Bladder dysfunction	2
Duration of surgery (mins)	101.3 ± 9.2
Blood loss (ml)	13.3 ± 3.9
Time in bed (h)	18.0 ± 7.0
Hospitalization time (days)	4.5 ± 1.3
Follow-up period (months)	14 ± 4.7

SD, standard deviation; *n*, number of patients.

The inclusion criteria were as follows: (1) TLDH diagnosis; (2) consistent symptoms, signs, and imaging findings; (3) complaints of a leg or thoracic radicular pain with or without low back pain, lower limb numbness with or without weakness, and walking difficulty, which cause severe suffering for patients; (4) conservatively treated for >3 months with limited therapeutic effect or no therapeutic effect; (5) learning the details of the procedure, including the surgical mechanism, possible clinical results, potential risks, and complications; and (6) ≥9-month follow-up postoperatively.

Exclusion criteria were patients (1) with complete cauda equina syndrome; (2) with dynamic instability or spondylolisthesis; (3) with anesthesia or medical conditions contraindicated for surgery; and (4) who were not cooperative.

### Surgical technique

We performed all operations using the Endo-surgi Plus system or Endo-surgi Standard system (Shanghai Maoyu Medical [Group] Co., LTD, China) with or without an endoscopic high-speed bur or piezosurgery, depending on surgical necessity. Tranexamic acid was used preoperatively to prevent bleeding (11). Nerve function monitoring was used to prevent intraoperative nerve injury as in our previous study (11).

#### Skin marking and placement of working cannula

**Figure 1:**
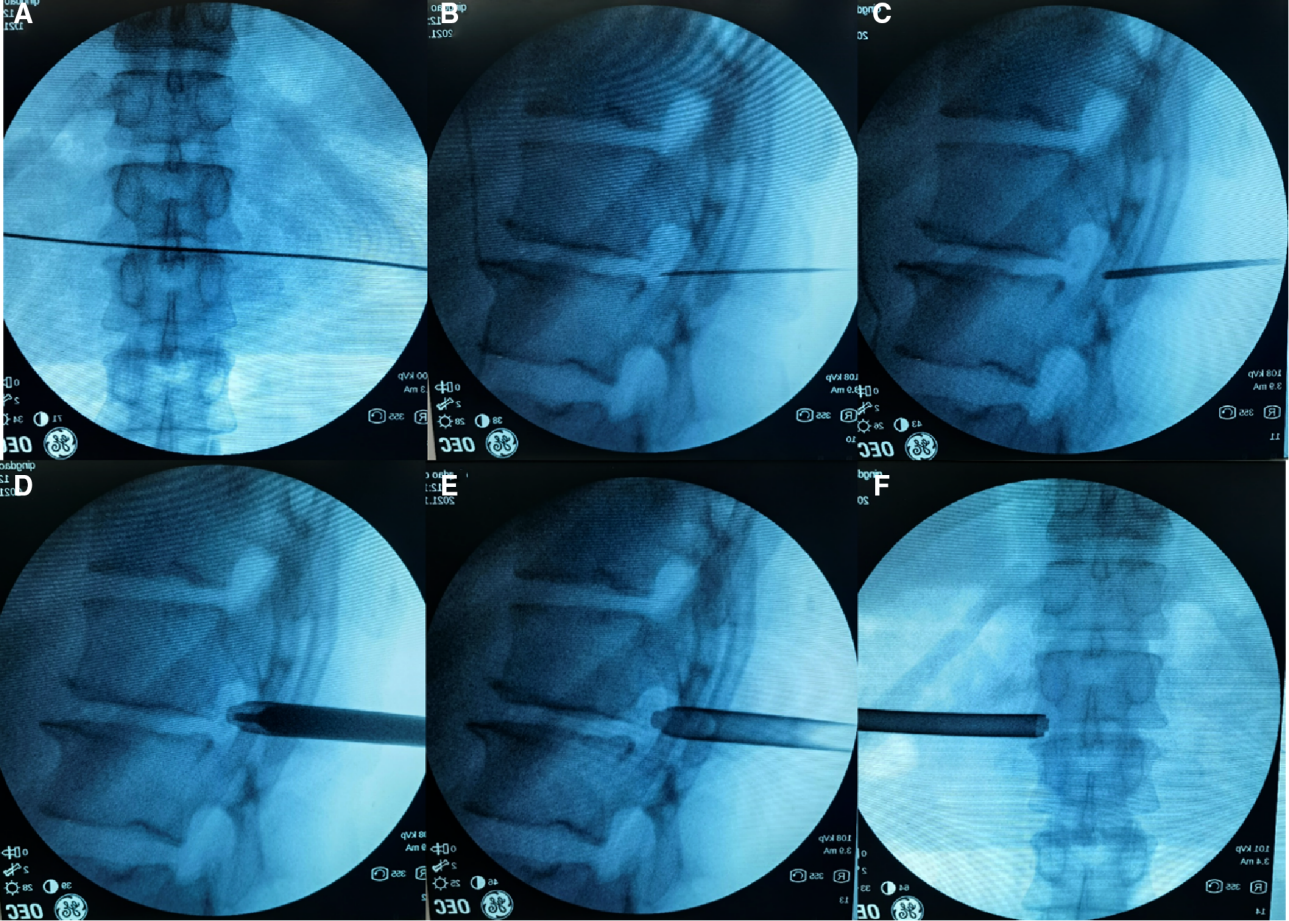
The procedure of establishing the working cannula with C-arm fluoroscopy assistance. (**A**) The location of segment to be operated. (**B**) The 18 G puncture needle inserted onto the lateral side of superior articular process. (**C**) The primary guide rod introduced through the guide wire. (**D**) The *U*-shaped working cannula introduced through the second guide rod. (**E,F**) The final location of the *U*-shaped working cannula under the anteroposterior and lateral view of C-arm fluoroscopy.

All patients were placed in the prone position on a radiolucent table after general anesthesia. The operation table was adjusted to enlarge the intervertebral foramen. The disc herniation segment was located under C-arm fluoroscopy (Figure 1A), and the puncture point approximately 6–8 cm lateral to the midline and tilted 10°–15° toward the cranial end was marked. An 18-G puncture needle was inserted onto the lateral side of the superior articular process (SAP) under C-arm fluoroscopy (Figure 1B) after disinfection and draping. The puncture needle was withdrawn after the guide wire was put into the needle, and the primary guide rod was introduced (Figure 1C) through the guide wire after making an incision. Then, a second guide rod and *U*-shaped working cannula were sequentially introduced (Figure 1D). The appropriate and safe location to avoid dura sac injury was confirmed with C-arm fluoroscopy, including the beveled end of the working channel not exceeding the line between the midpoints of pedicles on the same side in the anteroposterior view, and the beveled end of the working channel not exceeding the posterior edge of the vertebral body in the lateral view (Figures 1E,F).

#### Endoscopic procedure

**Figure 2:**
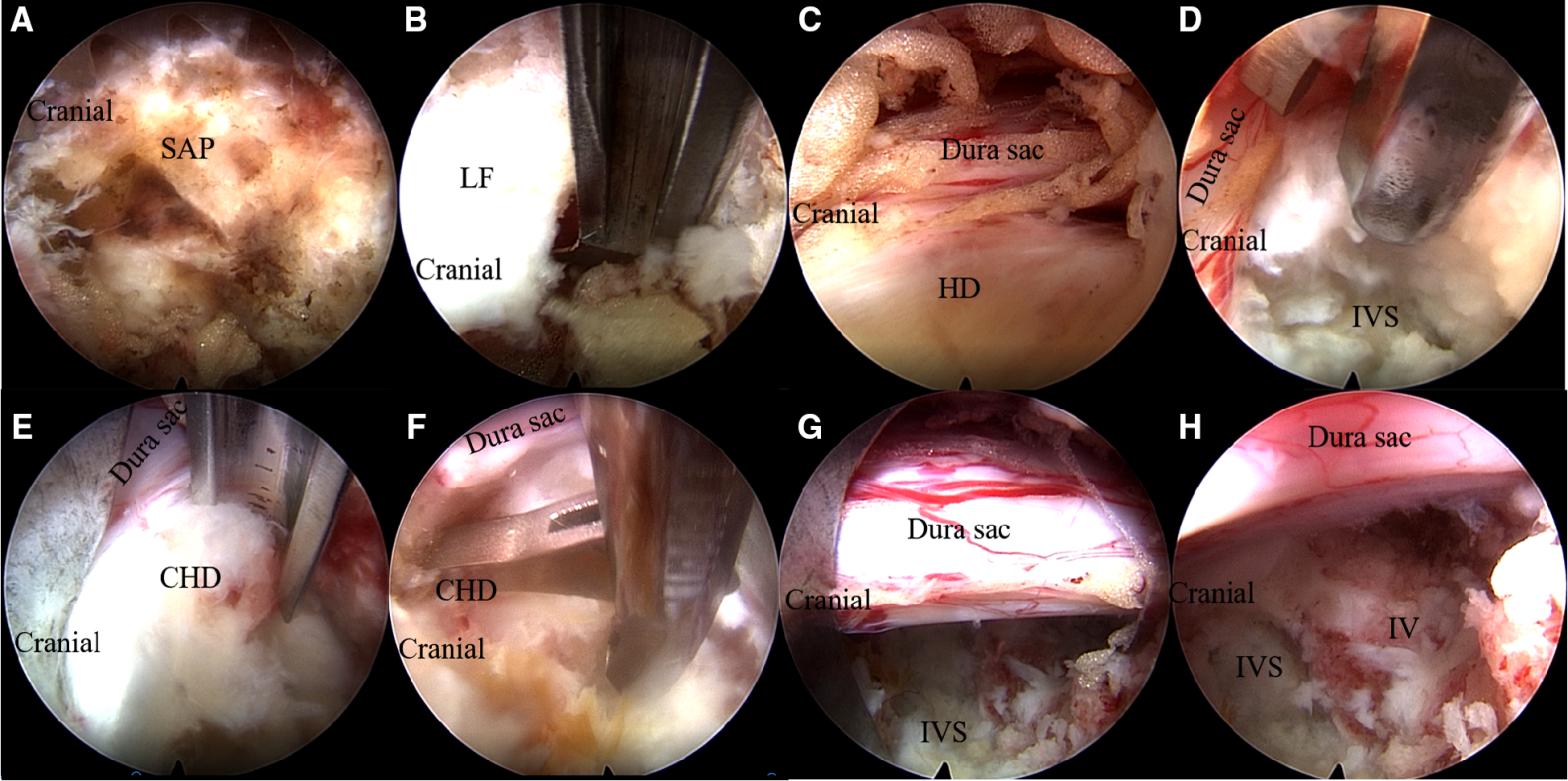
Main surgical procedures under endoscope. (**A**) The exposure of SAP and resection of SAP with a fully visualized trepan. (**B**) The ligamentum flavum ventral to the articular processes was resected with a Kerrison rongeur. (**C**) Exposure of the herniated disc and compressed dura sac. (**D**) Resection of the degenerated nucleus pulposus in the intervertebral space and the intervertebral disc protruded into the spinal canal with punch forceps. (**E**) Removal of the calcified herniated disc and ossified posterior longitudinal ligament at the posterior margin of the caudal vertebra with a chisel. (**F**) Removal of the calcified herniated disc at the posterior margin of the cranial vertebra with punch forceps. (**G,H**) Complete decompression of dura sac at the intervertebral space level and caudal side. SAP, superior articular process; LF, ligamentum flavum; HD, herniated disc; CHD, calcified herniated disc; IV, inferior vertebra; IVS, intervertebral space.

A T-shaped working cannula and an endoscope were introduced into the U-shaped cannula in sequence. The soft tissue in the foramen was cleaned to expose the anatomical structure of the foramen after complete hemostasis. Then, the SAP was exposed and partly resected with a fully visualized trepan or a Kerrison rongeur, or endoscopic high-speed bur, depending on the surgical need (Figure 2A). Next, part of the ligamentum flavum ventral to the articular processes was resected (Figure 2B) to expose the herniated disc and compressed dural sac (Figure 2C). The degenerated nucleus pulposus in the intervertebral space and the hump of the intervertebral disc protruding into the spinal canal were sequentially removed with punch forceps and grasping forceps to decompress the dural sac and nerve ventrally after the annulus fibrosus incision (Figure 2D). Afterward, the calcified disc or ossified posterior longitudinal ligament or osteophyte at the posterior margin of the caudal vertebra and cranial vertebra was sequentially chiseled away with a chisel or resected with punch forceps (Figures 2E,F). This sequence reduced the incidence of neck pain due to water pressure for patients under local anesthesia. The hypertrophic ligamentum flavum on the dorsal side of the dural sac was further resected to obtain adequate dural sac and nerve decompression after the elevated dural sac returned. Next, complete hemostasis was performed using radiofrequency electrodes. Satisfactory decompression was obtained (Figures 2G,H) and then the endoscope and the working cannula were withdrawn. Finally, the incision was sutured without placing a drainage tube.

### Outcome assessment

All included patients were evaluated preoperatively, at 1 day and 3, 6, and 12 months postoperatively or at last follow-up. Clinical outcomes were evaluated with the modified Japanese Orthopedic Association score (m-JOA) (12). The degree of low back and leg or thoracic radicular pain was evaluated with a visual analog scale (VAS). The modified MacNab criteria were used to assess clinical effectiveness 12 months postoperatively or the last follow-up (13). All patients were evaluated using magnetic resonance imaging (MRI) and computed tomography (CT) before discharge and at least once during the follow-up.

### Statistical analysis

We used Statistical Package for the Social Sciences (version 24.0; SPSS Inc., Chicago, IL, United States) for all clinical data statistical analyses. All data of pre- and postoperative VAS and m-JOA scores were expressed as mean ± standard deviation and were analyzed with the paired *t*-test if the data were normally distributed or were analyzed with Wilcoxon signed-rank test. Statistical significance was set at *P*-values of <0.05.

## Results

### Demographic characteristics and summary of primary clinical manifestations

This study included 12 patients, including 5 with soft disc herniation and 7 with disc herniation combined with calcification, according to the inclusion and exclusion criteria. Table 1 shows the patient demographic characteristics. Table 2 shows the preoperative primary clinical manifestations and imaging features of the 12 patients.

**Table 2:** Summary of preoperative primary clinical manifestations and imaging features of the 12 patients who were treated with fully endoscopic transforaminal discectomy and ventral decompression surgery.

Case No.	Age (years)	Sex	Location	Primary clinical manifestations	Soft/calcified (or with other type of calcification)
1	36	M	T12–L1	Leg pain, paresthesia in lower limb, neurogenic claudication	Calcified (OPLL)
2	40	F	T12–L1	Low back pain, leg pain, paresthesia in lower limb, neurogenic claudication	Calcified (OPLL)
3	63	M	T11–L2	Low back pain, thoracic radiculopathy, lower limb weakness	Soft
4	70	M	L1–2	Low back pain, leg pain, paresthesia in lower limb, neurogenic claudication	Calcified
5	42	M	T12–L1	Leg pain, paresthesia in lower limb, neurogenic claudication	Calcified (EPO)
6	38	F	T12–L1	Low back pain, leg pain paresthesia in lower limb, neurogenic claudication	Soft
7	40	M	T11–12	Leg pain, paresthesia in lower limb, neurogenic claudication	Calcified (EPO)
8	64	F	L1–L2	Low back pain, leg pain, paresthesia in lower limb, neurogenic claudication	Soft
9	62	F	T11–L2	Low back pain, thoracic radiculopathy paresthesia in lower limb, lower limb weakness	Soft
10	44	M	T12–L1	Low back pain, thoracic radiculopathy, lower limb weakness, bladder dysfunction	Calcified
11	71	F	T11–L2	Low back pain, thoracic radiculopathy, lower limb weakness	Calcified
12	66	M	L1–2	Low back pain, thoracic radiculopathy, lower limb weakness, bladder dysfunction	Soft

F, female; M, male; OPLL, ossification of posterior longitudinal ligament; EPO, endplate osteophyte.

### Clinical results

The excellent and good rate of patients evaluated with the modified MacNab criteria was 83.3%. Two patients presented fair results and occasionally demanded pain medication or physical therapy. Table 3 shows the detailed results. VAS scores for both low back pain and leg or thoracic radicular pain improved, with more significant improvement in the latter. Additionally, m-JOA showed significant improvement postoperatively than preoperatively. Significant differences were found in the preoperative and postoperative scores of m-JOA, low back pain VAS, and leg or thoracic radicular pain VAS at different time points (1 day and 3, 6, and 12 months postoperatively or at last follow-up) (Table 4). Moreover, Table 5 shows the major outcome preoperatively and 12 months postoperatively or last follow-up.

**Table 3:** Modified MacNab outcomes of 12 months after operation or at last follow-up (*n* = 12).

Outcomes	Description	*n* (%)
Excellent	Complete relief of symptoms	6 (50)
Good	Marked improvement but occasional pain	4 (33.3)
Fair	Improved functional capacity and the need for pain medications	2 (16.7)
Poor	Unimproved symptoms or worsening	0 (0)

*n*, number of patients.

**Table 4:** Mean change of outcome measurement (mean ± SD).

Outcome measurement	Pre-op	Before discharge	3-month post-op	6-month post-op	12-month post-op or last follow-up
Low back pain VAS	5.8 ± 1.5	4.2 ± 1.2*	2.8 ± 0.9*	2.2 ± 0.9*	2.0 ± 0.9*
Leg or thoracic radicular pain VAS	6.5 ± 1.4	3.9 ± 0.8*	2.5 ± 0.5*	1.3 ± 0.5*	1.3 ± 0.5*
m-JOA	7.5 ± 1.2	7.8 ± 0.9	9.0 ± 0.7*	9.3 ± 0.9*	10.0 ± 0.7*

*P < 0.05 versus preoperative data.

VAS, visual analog scale; SD, standard deviation; m-JOA, modified Japanese orthopedic association score; op, operation.

**Table 5:** Operation time, blood loss, time in bed, hospital stay time, follow-up period, and pre- and postoperative m-JOA and VAS assessed 12 months postoperatively or at last follow-up.

Case No.	Op time (mins)	Blood loss (ml)	Time in bed (h)	Hospital stay time (days)	Follow-up period (months)	m-JOA		Low back pain VAS		Leg or thoracic radicular pain VAS	
						Pre-op	Post-op	Pre-op	Post-op	Pre-op	Post-op
1	125	20	12	3	24	6	10	3	1	4	1
2	104	15	24	3	18	9	11	8	2	9	1
3	100	10	16	4	18	8	10	5	2	6	1
4	110	10	20	6	18	7	9	7	3	7	2
5	98	15	14	5	15	6	10	4	2	5	1
6	92	20	15	3	12	9	11	7	0	7	1
7	105	10	19	5	12	7	9	6	2	8	1
8	102	10	12	4	12	6	10	4	2	5	1
9	96	15	22	4	12	8	10	6	2	6	1
10	94	10	12	4	9	9	11	7	2	7	2
11	95	15	36	7	9	7	9	7	4	8	2
12	94	10	14	6	9	8	10	6	2	6	1

m-JOA, modified Japanese orthopedic association score; VAS, visual analog scale; op, operation.

### Complications

This study revealed a 16.7% incidence of minor complications, where two patients experienced transient lower limb dysesthesia postoperatively. The dysesthesia was relieved upon the 3-month follow-up visit. No severe complications, such as lung injury, pleura injury, viscera injury, spinal cord injury, nerve injury, dural tear, and cerebrospinal fluid (CSF) leakage occurred.

### Representative case

**Figure 3:**
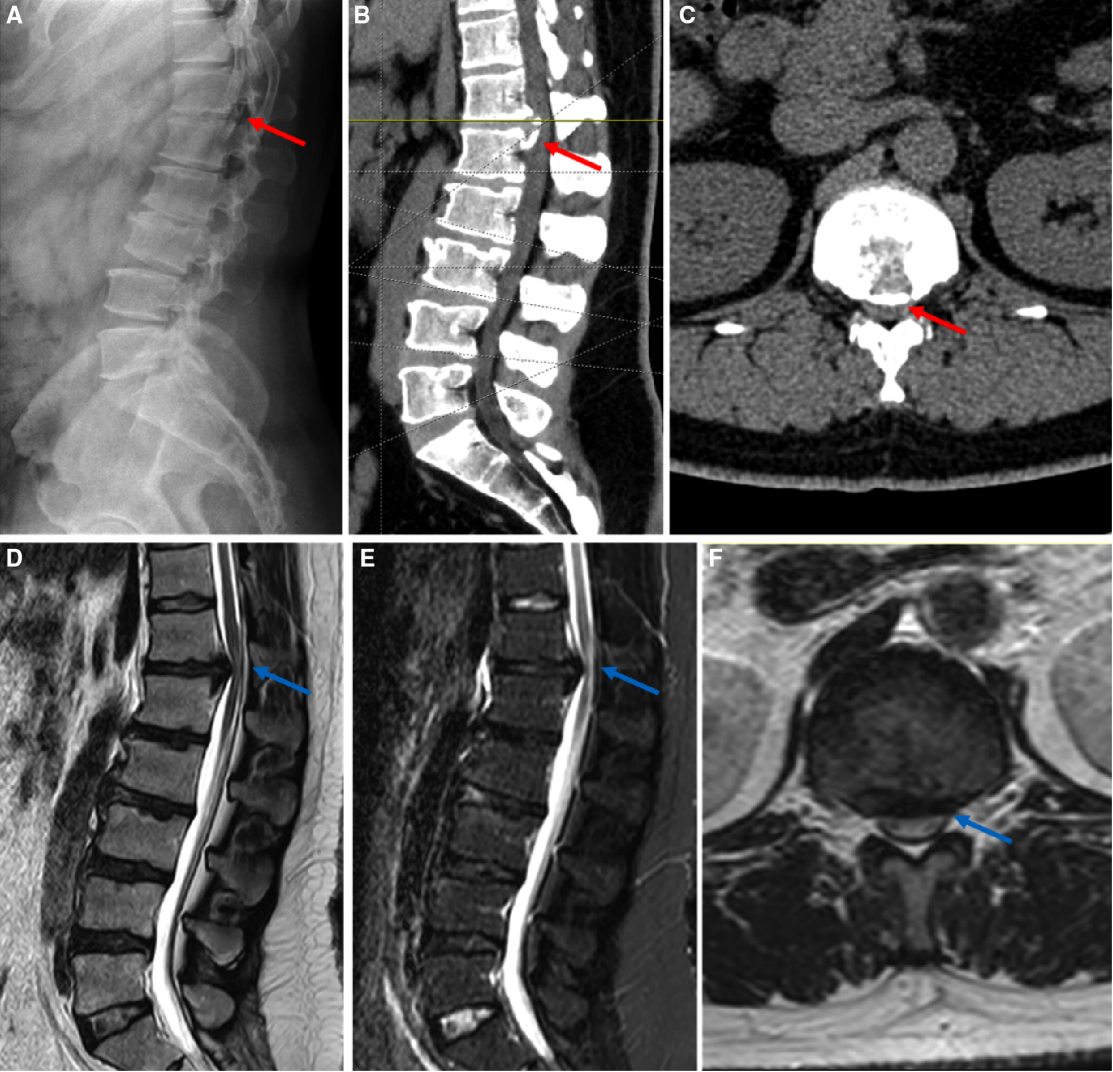
Preoperative imaging of the typical patient with disc herniation at the T12–L1 level. A 38-year male patient diagnosed with TLDH with calcification at the T12–L1 level underwent PETD under general anesthesia. (**A–C**) Preoperative x-ray and CT. The red arrow in (**A–C**) highlighted calcified herniated disc and ossified posterior longitudinal ligament. (**D–F**) Preoperative MRI. The blue arrows in (**D–F**) highlighted severe compression of spinal cord and the normal signal of cerebrospinal fluid cannot be seen. (**A,B,D,E**) sagittal view; (**C,F**) axial view; TLDH, thoracolumbar junction disc herniation; PETD, percutaneous endoscopic transforaminal discectomy; CT, computed tomography; MRI, magnetic resonance imaging.

**Figure 4:**
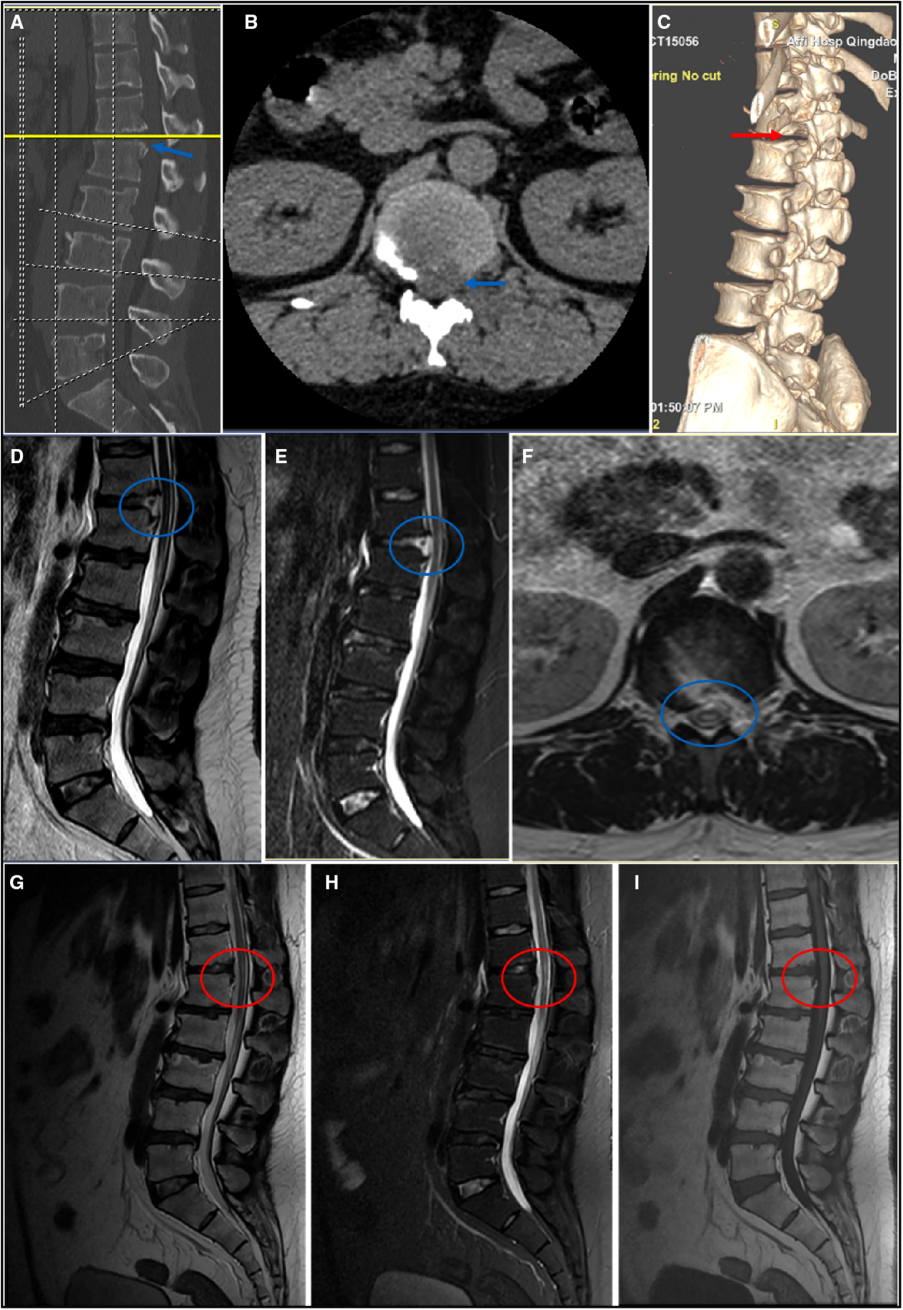
Postoperative imaging of the typical patient with disc herniation at the T12–L1 level. (**A–C**) CT at 1 day postoperatively. The blue arrow shows sufficient removal of calcific herniated disc. The red arrow shows that the SAP is partly resected and the stability of the spine is preserved. (**D–F**) MRI at 1 day postoperatively. The blue circle shows complete decompression of dura sac with a small amount of fluid signal in the ventral side of dura sac. (**G–I**) MRI at 6 months postoperatively. The red circle shows perfect decompression of dura sac and normal signal of cerebrospinal fluid surrounding the dura. (**A,D,E,G–I**) sagittal view; (**B,F**) axial view; (**C**) three-dimensional reconstruction. SAP, superior articular process; CT, computed tomography; MRI, magnetic resonance imaging.

A 38-year-old male patient suffered from low back pain and both lower limb weakness, combined with intermittent claudication for >1 year. His symptoms gradually worsened, and his VAS score for low back pain was 6 out of 10. The distance of intermittent claudication is approximately 300 m. Physical examination demonstrated bilateral lower leg and feet hypoesthesia, which is more severe on the left side, as well as decreased muscle power of tibialis anterior and extensor hallucis longus to grades 3 and 4 on the left and right sides, respectively. The dynamic lumbar radiography showed no segmental instability at the T12–L1 level. MRI and CT revealed severe central disc herniation combined with calcification and ossification of posterior longitudinal ligament at the T12–L1 level (Figure 3). Thus, we performed PETD at the T12–L1 level. The patient got out of bed and ambulated approximately 16 h postoperatively. His VAS score for low back pain decreased from 6 to 4, and his bilateral muscle power of the tibialis anterior and extensor hallucis longus recovered partly postoperatively. MRI and CT at 1 day postoperatively revealed sufficient decompression (Figures 4A–F). His back pain completely disappeared 3 months postoperatively. Additionally, his bilateral muscle power of the tibialis anterior and extensor hallucis longus recovered to grade 5 at 6 months postoperatively, and MRI showed perfect dural sac decompression and normal CSF signals surrounding the dura (Figures 4G–I).

## Discussion

Generally, the anatomical structure of the thoracolumbar junction zone is different from the lower lumbar vertebrae. First, the spinal cord transitions to the cauda equina in the thoracolumbar junction zone (1). Second, the dural sac diameter in the thoracolumbar junction zone is larger than in the lower lumbar spine (6). Third, the space between the 2 pars interarticularis, as well as the interlaminar window, gets smaller, and the inferior edge of the lamina covers more of the intervertebral space (6). Therefore, the clinical manifestations of disc herniation in this region are different from the thoracic and lower lumbar vertebrae (1). Additionally, performing a discectomy and ventral decompression surgery for disc herniation in this region is more challenging than in the lower lumbar vertebrae. Therefore, selecting the appropriate surgical method to remove herniated discs in the thoracolumbar junction zone is very important.

Conventional open thoracic discectomy and decompression surgery incurs great trauma, has a high rate of complications, and always demands additional internal fixation (7, 14). PETD was widely accepted by surgeons and patients due to its advantages of being less invasive, rapid recovery, less bleeding, short hospital stay, and low cost, with the popularization of minimally invasive concepts and advances in endoscopic techniques (15, 16). The excellent as well as good outcomes and the advantages of full-endoscopic spine surgery have been proven for the treatment of herniated discs and stenoses in the lumbar and cervical vertebrae (11, 17, 18). Additionally, the next step after mastering lumbar and cervical endoscopic spinal surgery is managing the thoracic pathology with the full-endoscopic technique (19). Recently, many surgeons worldwide tried various minimally invasive surgery techniques, such as surgery-transforaminal lumbar interbody fusion, video-assisted thoracoscopic surgery, microendoscopic surgery, and full-endoscopic surgery, to treat thoracic pathology (4, 9, 20–22).

Few studies were conducted to investigate TLDH as a specific type of disc herniation due to the low incidence rate of TLDH (4, 6, 22). This retrospective study regarded the disc herniation at the thoracolumbar junction zone (T11–12, T12–L1, and L1–2) as a special entity of disc herniation and reported the early clinical outcomes of 12 patients with TLDH and treated with PETD. Encouragingly, our cases revealed 83.3% excellent and good rates with the modified MacNab criteria. A review reported that excellent or good outcomes were achieved for full-endoscopic procedures in a mean of 81% of patients with thoracic pathology (range 46%–100%) (23). Ahn et al. reported 77.8% excellent and good rates of L1–L2 and the L2–L3 levels treated with PETD (24). The clinical efficacy of this article was comparable with the published results (23, 24). Gao et al. reported 11 cases of symptomatic thoracic disc herniation treated with a full-endoscopic transforaminal ventral decompression technique (9). The mean m-JOA improved from 7.4 preoperatively to 10.2 at last follow-up (9). Additionally, the mean m-JOA of six thoracic disc herniation cases reported by Guo et al. improved from 4.4 preoperatively to 6.6 1 year postoperatively (25). The mean m-JOA in the present study improved from 7.5 preoperatively to 10.0 12 months postoperatively or at last follow-up, which was similar to previous studies (9, 25). A mean VAS improvement from 5.8 to 2.0 for low back pain and 6.5 to 1.3 for leg or thoracic radicular pain in this study was close to the study by Choi et al. (26). Furthermore, postoperative MRI in all patients showed sufficient ventral spinal cord decompression and unobstructed cerebrospinal fluid circulation in the spinal canal.

Ruetten et al. reported a 20% complication rate, of which 8% were severe complications, including one epidural hematoma without revision and one myelopathy deterioration (27). However, severe complications were not documented in the present study and studies by Guo et al. (25) and Gao et al. (9). This is because of the small sample size of our study and careful manipulation as well as nerve function monitoring in operation. Two patients complained of unsatisfactory relief of their low back pain, which could be relieved with nonsteroidal analgesics and physical therapy.

Our study revealed satisfactory clinical outcomes without severe complications because of the following four main aspects. First, the beveled end of the working cannula was not inserted into the spinal canal before introducing the endoscope, and foraminoplasty was performed with a fully visualized trepan under the endoscope, not only enlarging the foramen according to decompression requirement but also avoiding the dural sac, as well as nerve injury. Second, the diameter of the dural sac in the thoracolumbar junction region is larger and the diameter of the spinal canal is smaller than the lower lumbar vertebrae (6). Thus, the epidural space is small, and the surrounding anatomical environment lacks sufficient buffer space. Therefore, removing the disc in the intervertebral space as indirect “box-shaped decompression” described by Ruetten et al. before direct removal of herniated disc compressing the dural sac, avoiding spinal cord injury, especially for herniated disc combined with calcification or endplate osteophyte or local ossification of posterior longitudinal ligament, is very important (27). Third, the calcific herniated disc is more difficult to remove than the soft herniated disc. Thus, after removing the herniated disc on the intervertebral level, the direction of the working cannula needs to be adjusted to meticulously resect the calcific herniated disc (or osteophyte or local ossification of the posterior longitudinal ligament) at the posterior margin of the caudal and cranial vertebra in sequence. This sequence can help avoid or at least reduce the incidence of neck pain due to water pressure in patients under local anesthesia. Fourth, tranexamic acid was used preoperatively to reduce bleeding and nerve function monitoring was used intraoperatively to prevent spinal cord and nerve injury in all the operations, as in our previous study (11).

Foraminoplasty has become increasingly safe with advances in the full-visualized trephine technique. It also enables patients to receive percutaneous endoscopic lumbar discectomy under general anesthesia. General anesthesia could reduce patients’ intraoperative pain and tension and significantly improve patients’ surgical experience (28). Additionally, general anesthesia provides surgeons with the opportunity to focus more on the operation and shorten the operative time without worrying about the patient's intraoperative feelings during the operation. From our point of view, full-endoscopic discectomy under general anesthesia is safe and does not significantly increase the incidence of complications. However, postoperative complications, such as nerve root injury in 10% of patients and nausea, vomiting, dizziness, and drowsiness in 15% of patients under general anesthesia, were observed in another study (29).

Our study limitations are obvious. The sample size was small; thus, our conclusion is less persuasive. Additionally, potential risks and complications are associated with this technique. Furthermore, this observational study had early results; therefore, prospective randomized controlled studies with larger sample sizes and long-term follow-up should be conducted in the future to obtain more convincing conclusions.

## Conclusion

Fully endoscopic transforaminal discectomy and ventral decompression under general anesthesia is a safe, feasible, effective, and minimally invasive method for treating herniated discs with or without calcification at the thoracolumbar junction zone.

## Data Availability

The raw data supporting the conclusions of this article will be made available by the authors, without undue reservation.
